# *KCNV2*-associated retinopathy: genotype–phenotype correlations – *KCNV2* study group report 3

**DOI:** 10.1136/bjo-2023-323640

**Published:** 2023-10-18

**Authors:** Thales A C de Guimaraes, Michalis Georgiou, Anthony G Robson, Kaoru Fujinami, Ajoy Vincent, Fadi Nasser, Samer Khateb, Omar A Mahroo, Nikolas Pontikos, Maurício E Vargas, Alberta A H J Thiadens, Emanuel R de Carvalho, Xuan-Than-An Nguyen, Gavin Arno, Yu Fujinami-Yokokawa, Xiao Liu, Kazushige Tsunoda, Takaaki Hayashi, Belén Jiménez-Rolando, Maria Inmaculada Martin-Merida, Almudena Avila-Fernandez, Ester Carreño Salas, Blanca Garcia-Sandoval, Carmen Ayuso, Dror Sharon, Susanne Kohl, Rachel M Huckfeldt, Eyal Banin, Mark E Pennesi, Arif O Khan, Bernd Wissinger, Andrew R Webster, Elise Heon, Camiel J F Boon, Eberhard Zrenner, Michel Michaelides

**Affiliations:** 1 Institute of Ophthalmology, University College London, London, UK; 2 Moorfields Eye Hospital NHS Foundation Trust, London, UK; 3 Laboratory of Visual Physiology, Division of Vision Research, National Institute of Sensory Organs, Tokyo, Japan; 4 Department of Ophthalmology, Keio University School of Medicine, Tokyo, Japan; 5 Department of Ophthalmology and Vision Sciences, The Hospital for Sick Children, Toronto, Ontario, Canada; 6 Centre for Ophthalmology, University Hospital Tubingen Institute for Ophthalmic Research, Tubingen, Germany; 7 Department of Ophthalmology, Hadassah Medical Center, Jerusalem, Israel; 8 Genetic Eye Therapies, Ventura, California, USA; 9 Department of Opthalmology, Erasmus Medical Center Rotterdam, Rotterdam, The Netherlands; 10 Department of Ophthalmology, Amsterdam University Medical Centres, Amsterdam, The Netherlands; 11 Department of Ophthalmology, Leiden University Medical Center, Leiden, The Netherlands; 12 Department of Health Policy and Management, Keio University School of Medicine, Tokyo, Japan; 13 Department of Ophthalmology, The Jikei University School of Medicine, Tokyo, Japan; 14 Instituto de Investigacion Sanitaria de la Fundacion Jimenez Diaz, Madrid, Spain; 15 Center for Biomedical Network Research on Rare Diseases (CIBERER), Madrid, Spain; 16 Department of Ophthalmology, Massachusetts Eye and Ear, Harvard, Massachusetts, USA; 17 Department of Ophthalmology, Oregon Health & Science University - Casey Eye Institute, Portland, Oregon, USA; 18 Eye Institute, Cleveland Clinic Abu Dhabi, Abu Dhabi, UAE; 19 Department of Ophthalmology, Cleveland Clinic Lerner College of Medicine of Case Western Reserve University, Cleveland, Ohio, USA

**Keywords:** Genetics, Imaging, Electrophysiology, Dystrophy, Retina

## Abstract

**Background/aims:**

To investigate genotype–phenotype associations in patients with *KCNV2* retinopathy.

**Methods:**

Review of clinical notes, best-corrected visual acuity (BCVA), molecular variants, electroretinography (ERG) and retinal imaging. Subjects were grouped according to the combination of *KCNV2* variants—two loss-of-function (TLOF), two missense (TM) or one of each (MLOF)—and parameters were compared.

**Results:**

Ninety-two patients were included. The mean age of onset (mean±SD) in TLOF (n=55), TM (n=23) and MLOF (n=14) groups was 3.51±0.58, 4.07±2.76 and 5.54±3.38 years, respectively. The mean LogMAR BCVA (±SD) at baseline in TLOF, TM and MLOF groups was 0.89±0.25, 0.67±0.38 and 0.81±0.35 for right, and 0.88±0.26, 0.69±0.33 and 0.78±0.33 for left eyes, respectively. The difference in BCVA between groups at baseline was significant in right (p=0.03) and left eyes (p=0.035). Mean outer nuclear layer thickness (±SD) at baseline in TLOF, MLOF and TM groups was 37.07±15.20 µm, 40.67±12.53 and 40.38±18.67, respectively, which was not significantly different (p=0.85). The mean ellipsoid zone width (EZW) loss (±SD) was 2051 µm (±1318) for patients in the TLOF, and 1314 µm (±965) for MLOF. Only one patient in the TM group had EZW loss at presentation. There was considerable overlap in ERG findings, although the largest DA 10 ERG b-waves were associated with TLOF and the smallest with TM variants.

**Conclusions:**

Patients with missense alterations had better BCVA and greater structural integrity. This is important for patient prognostication and counselling, as well as stratification for future gene therapy trials.

**WHAT IS ALREADY KNOWN ON THIS TOPIC**

- *KCNV2*-associated retinopathy is a rare form of autosomal recessive inherited retinal dystrophy. The electrophysiology, retinal imaging and clinical course of the disease have been well described in the literature. Genotype–phenotype correlations have not been elucidated to date.

**WHAT THIS STUDY ADDS**

- This study represents the largest attempt to establish genotype–phenotype in affected individuals. We provide evidence that individuals with two missense variants in trans had a better best-corrected visual acuity and more preserved structural integrity.

**HOW THIS STUDY MIGHT AFFECT RESEARCH, PRACTICE OR POLICY**

- Data from this study will arm clinicians with more informed tools for patient prognostication and counselling, as well as assist in patient stratification for future clinical trials.

## Introduction

*KCNV2*-associated retinopathy (cone dystrophy with supernormal rod responses; OMIM #610356) is a rare form of autosomal recessive (AR) inherited retinal disease (IRD) with a pathognomonic electroretinogram (ERG).^1^ ^, 2^ The *KCNV2* retinopathy study group is the largest multicentre retrospective investigation of affected individuals. Report No.1 of the study established an early-onset disease, outlined the characteristic ERG changes that were consistent with a largely stable retinal dysfunction across many decades, and reported 75 disease-causing variants, out of which 18 (37.3%) were novel.^2^ Report No.2 characterised the retinal architecture and structural changes in optical coherence tomography (OCT) and fundus autofluorescence (FAF) imaging cross-sectionally and with disease natural history, identifying a slowly progressive thinning of the outer nuclear layer (ONL) and disruption of the ellipsoid zone (EZ), with a large window for therapeutic intervention up to approximately 40 years of age.^3^ Genotype–phenotype correlations have not been studied to date in a large cohort of affected individuals.

Advances in molecular diagnostic techniques have improved our understanding of the genetic basis of IRDs.^4^ Due to the inherent clinical heterogeneity of IRDs, establishing genotype–phenotype correlations is a crucial step in counselling and clinical management of these disorders, as it carries direct prognostic implications.^5 6^ Genotype–phenotype correlations have been investigated for several genotypes, such as *ABCA4* (OMIM #601691),^6 7^ *USH2A* (OMIM #608400),^8^ *RPGR* (OMIM #312610),^9 10^ *RP1* (OMIM #603937),^11^ *RS1* (OMIM #300839)^5 12^ and *CRB1* (OMIM #604210).^13 14^ These are among the most common genes identified in individuals affected with IRD.^4 15^ A range of structural and functional abnormalities have been reported for *KCNV2* retinopathy, however genotype–phenotype correlations have not been elucidated, given the relative rarity and the phenotypic diversity of the disease.^16–19^ Recently, a report found no uniformly presenting phenotype among 14 Arabian Peninsula individuals with a founder mutation (c.427G>T; p.Glu143*).^20^

*KCNV2*-retinopathy is a potential target for gene supplementation therapy, and establishing genotype–phenotype correlations is important for identifying potential candidates for human clinical trials and to advise patients regarding visual prognosis. Here, we present Report No.3, which aims to explore genotype–phenotype correlations for the disease.

## Material and methods

### Patient identification

The *KCNV2* study group is an international retrospective study of patients with *KCNV2*-associated retinopathy. In the current report, we included patients molecularly confirmed to have biallelic *KCNV2* variants. We excluded patients in whom retinal imaging and phenotypic details such as age of onset, best-corrected visual acuity (BCVA) and symptomatology, were not available. Fundus examination is mainly descriptive in nature; hence, the authors chose to not include it herein. The methodology for each test is described in the first two reports.^2 3^

### Genetic variant grouping

Here, we defined loss-of-function as canonical splice site, nonsense and frameshift variants, and large deletions (ie, structural variants). The patients were then grouped into three categories based on the molecular variants identified: (i) two loss-of-function (TLOF), (ii) one missense and one loss-of-function (MLOF) and (iii) two missense variants (TM). With the purpose of identifying if sequence variants in specific protein domains are associated with a more severe phenotype, we will also be alluding in each section to the presence of missense variants in highly conserved regions—such as N-terminal A and B box and the pore-forming loop structure (P loop)—in either TM and/or MLOF groups.

### Clinical and imaging data

For this analysis, the authors associated baseline characteristics and their rate of change, including: BCVA (in Logarithm of the Minimal Angle of Resolution, (LogMAR)), age of onset (in years), quantitative (ONL thickness, EZ width (EZW) loss and area of decreased autofluorescence (DAF)) and qualitative retinal imaging, and electrophysiology, to investigate trends and possible associations. Quantitative analysis was only performed in patients seen in a single centre (Moorfields Eye Hospital, London, UK). Three distinct macular FAF features were previously identified: (1) centrally increased autofluorescence, (2) DAF and (3) perimacular ring of increased autofluorescence; whereas five distinct FAF groups were identified based on combinations of those features: (1) group 1: negative for all three features, (2) group 2: increased central AF, (3) group 3: perimacular ring and centrally increased AF, (4) group 4: perimacular ring without centrally increased AF, (5) group 5: DAF and perimacular ring.^3^ FAF interocular symmetry was established in Report No.2 (3), hence only retinal imaging parameters of right eyes were used here.

### Electrophysiology

Pattern and full-field ERG (PERG; ERG) assessments were performed on 30 patients and incorporated the International Society for Clinical Electrophysiology of Vision (ISCEV) standards.^21 22^ The PERG P50 component was used to assess macular function and the ERG used to assess generalised rod and cone system function. The ERG data were compared with reference values from a control group of subjects without retinal or visual pathway disease (age range: 10–79 years).^2^

### Statistical methods

Statistical analysis was performed with the aid of the software GraphPad Prism V.9 (GraphPad Software; San Diego, California, USA). Parametric and non-parametric tests were employed, as well as correlation parameters (either Pearson or Spearman). Significance of all statistical tests was set at p<0.05 and D’Agostino-Pearson test (omnibus K2) was used to determine normality for all variables. Descriptive statistics was used in patients identified with variants in conserved residues.

## Results

### Group classification

**Figure 1:**
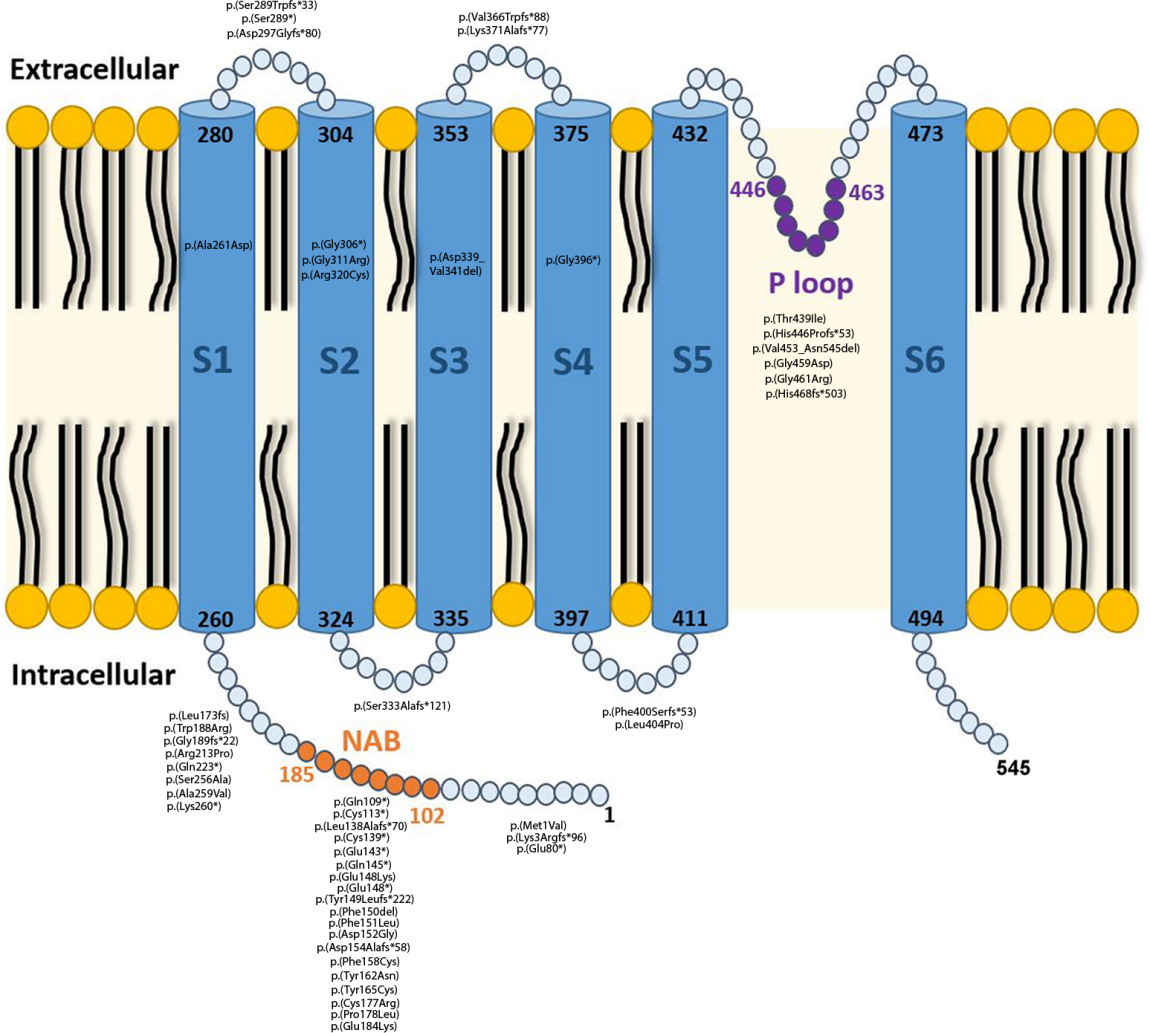
Protein representation and identified variants. Variants found in the affected individuals reported herein according to protein location. The alpha-subunit of the potassium channel (Kv8.2) encoded by *KCNV2* consists of: (i) a highly conserved tetramerisation domain; N-terminal A and B box (NAB) that facilitates interaction between compatible alpha-subunits; (ii) 6 transmembrane domains (S1–S6);(iii) extracellular and intracellular loop segments and (iv) an ultra-conserved potassium selective motif in the pore-forming loop between S5-S6 (P loop).

In total, 92 patients met the inclusion criteria: (1) 55 patients were identified with TLOF variants (60%), (2) 23 with TM alterations (25%), out of whom 17 (74%) included at least one variant in a conserved domain and (3) 14 with a combination of MLOF variants (15%), 6 of whom (43%) had the missense alteration in a conserved domain. The variants found in this cohort are presented in figure 1.

### Disease onset and symptoms

**Figure 2:**
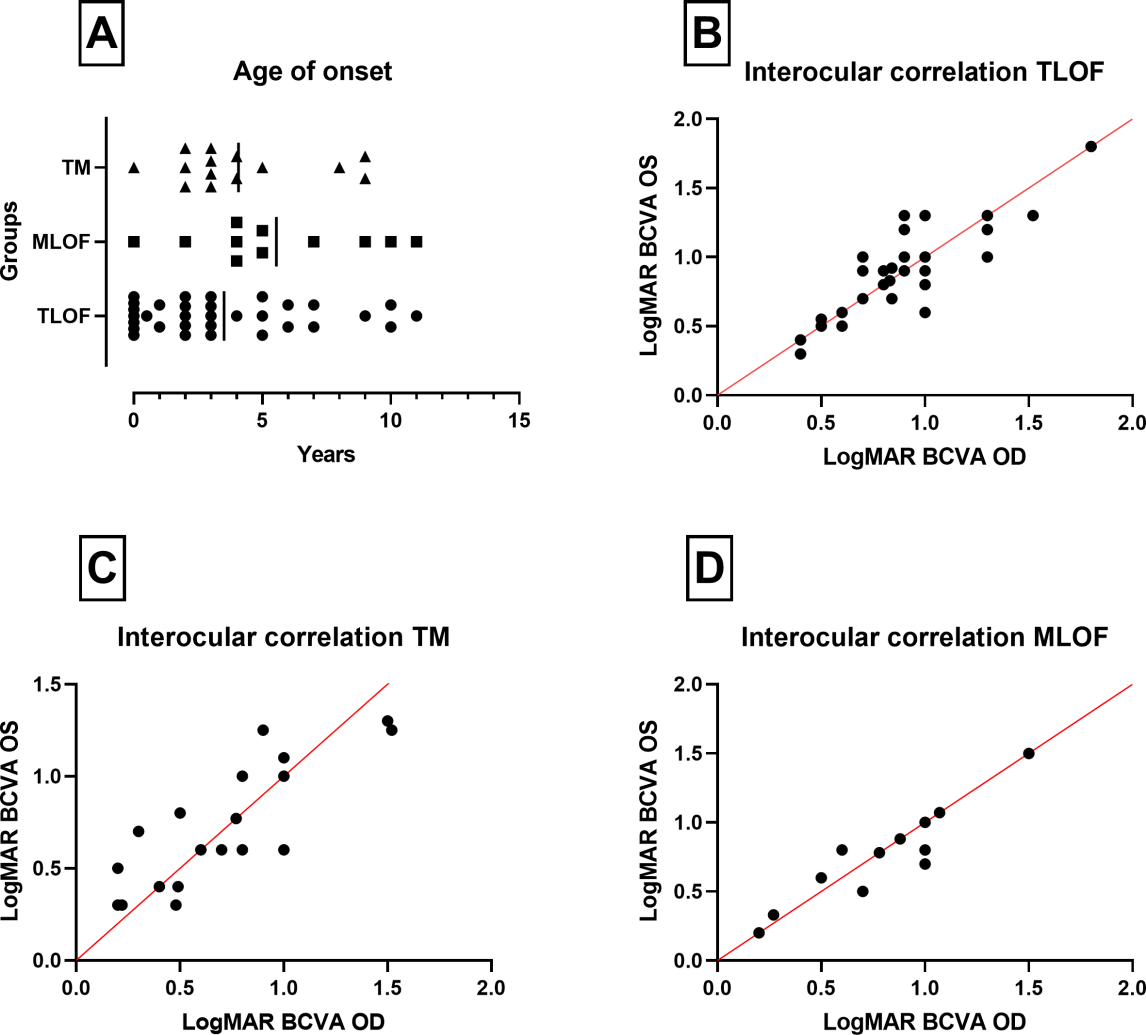
Variant grouping, age of onset and BCVA analysis. (A) Age of onset of patients in the three variant groups, TM (triangles), MLOF (squares) and TLOF (circles). The vertical line represents the mean of each group. The mean age of onset was earliest for TLOF. The interocular correlation of BCVA (Pearson correlation coefficient) was calculated for all groups, with a line of identity in red. The correlation was high in all three groups: (B) TLOF (B; r=0.86; p<0.0001), (C) TM (C; r=0.83; p<0.0001) and (D) MLOF (D; r=0.92; p<0.0001), which implies a high interocular symmetry from a functional perspective. BCVA, best-corrected visual acuity; MLOF, missense and one loss-of-function variant; TLOF, two loss-of-function variants; TM, two missense alterations.

In the TLOF group, 23.6% of patients (n=13) had disease onset in infancy, as opposed to 26% (n=6) in the TM group, of whom 5 had the missense alterations in conserved protein domains, and 28.5% (n=4) in the MLOF, all whom also had a conserved variant. In patients in whom the exact age of onset was known (n=57), the mean age of onset (range; ±SD), for TLOF, TM and MLOF groups was 3.51 (0–11; ±0.58), 4.07 (0–9; ±2.76) and 5.54 (0–11; ±3.38) years, respectively (figure 2A), which was not significantly different (one-way analysis of variance (ANOVA); F ratio=1.661; p=0.19). Eighteen patients had the common c.1381G>A (p.Gly461Arg) variant—that was previously identified as a mutational hotspot—combined with another missense variant (TM group) or in trans with a loss-of-function variant (MLOF), 12 of whom had the exact age of onset known.^1^ The mean age of onset of patients with this variant on at least one allele was 4 years (range=0–9; ±2.66). Most patients in our cohort also reported additional symptoms, such as nyctalopia, photophobia or colour vision difficulties, with 83% (n=46) of TLOF patients having one or more associated symptoms, as opposed to 61% (n=14) and 86% (n=12) for the TM and MLOF groups, respectively. Table 1 summarises the findings described in this section.

**Table 1:** Mean, range and SD of main parameters analysed

Parameter (range; SD)	TLOF group	MLOF group	TM group	P value
Mean age of onset (years)	3.51 (0–11; ±0.58)	5.54 (0–11; ±3.38)	4.07 (0–9; ±2.76)	0.19
Mean age at baseline visit (years)	21.3 (1–68; ±15.7)	23.1 (4–51; ±14.8)	16.9 (4–53; ±16.3)	0.45
Mean baseline LogMAR BCVA OD	0.89 (0.4–1.8; ±0.25)	0.81 (0.2–1.5; ±0.35)	0.67 (0.2–1.52; ±0.38)	0.03
Mean baseline LogMAR BCVA OS	0.88 (0.3–1.8; ±0.26)	0.78 (0.2–1.5; ±0.33)	0.69 (0.3–1.3; ±0.33)	0.035
Mean ONL thickness at baseline (µm)*	37.07 (17–70; ±15.2)	40.67 (20–59; ±12.53)	40.38 (14–68; ±18.67)	0.85
Mean annual rate of ONL thickness change (µm)*	1.29 (−2.5 to −0.5; ±0.9)	1.0 (−2.5 to 0; ±1.08)	1.29 (−3.0 to 0; ±1.35)	0.91
Mean EZ width loss at baseline (µm)*	2051 (538–4318; ±1318)	1314 (437–2359; ±965)	1181†	0.32
Mean annual rate of EZ width loss change (µm)*	197.6 (10.5–810.5; ±259)	58.88 (35.5–90; ±22.8)	29†	0.14

The p values for EZ width loss at baseline and mean annual rate of EZ width loss change are for an unpaired t-test between TLOF and MLOF groups, which is otherwise related to one-way ANOVA between the three groups.

*Only right eye included.

†Only one patient had measurable EZ width in the TM group, indicating greater structural integrity.

ANOVA, analysis of variance; BCVA, best-corrected visual acuity; EZ, ellipsoid zone; LogMAR, Logarithm of the Minimum Angle of Resolution; MLOF, one missense and one loss-of-function variant; OD, right eye; ONL, outer nuclear layer; OS, left eye; TLOF, two loss-of-function variants; TM, two missense variants.

### Visual acuity

There was variability in patient’s age at their first recorded BCVA. The mean age at the baseline visit (in years) in TLOF, TM and MLOF was 21.3 (range=1–68; SD±15.7), 23.1 (range=4–51; SD±14.8) and 16.9 (range=4–53; SD±16.3), respectively, which was not significantly different (one-way ANOVA; F ratio=0.8; p=0.45). The mean LogMAR BCVA for TLOF, TM and MLOF was (1) 0.89 (range=0.40–1.80; SD±0.25), 0.67 (range=0.20–1.52; SD±0.38) and 0.81 (range=0.20–1.50; SD=±0.35) for right eyes, and (2) 0.88 (range=0.30–1.80; SD±0.26), 0.69 (range=0.30–1.3; SD=±0.33) and 0.78 (range=0.20–1.50; SD±0.33) for left eyes, respectively, revealing significant correlation between eyes in all groups (p<0.0001; figure 2B–D). This difference in cross-sectional BCVA at baseline was found to be statistically significant in both right (one-way ANOVA; F ratio=3.63; p=0.03) and left eyes (one-way ANOVA; F ratio=3.46; p=0.035). Fifteen patients (mean age=21.6; range=4–51; SD±16.4) in either the MLOF and TM groups that had the variant c.1381G>A (p.Gly461Arg) on at least one allele, had a mean baseline BCVA of 0.77 (range=0.2–1.52; SD±0.43) in right and 0.71 (range=0.2–1.52; SD±0.42) in left eyes, which also revealed high interocular correlation (Pearson coefficient; r=0.95; p<0.0001).

### Optical coherence tomography

Seventy-nine patients had at least one good quality OCT at baseline and were therefore included. Fifty-three patients had a follow-up image after at least 24 months from baseline. Qualitatively, as identified in Report No.2, OCT grades 3, 4 and 5 had measurable EZ loss. OCT in either of these grades was found in 57% (n=27) of patients in the TLOF group. Group 5 (atrophy) was identified in more than half (n=15; 55%) of TLOF patients, and 50% (n=6) and 25% (n=5) in the MLOF and TM groups, respectively. Of the patients who had follow-up data, 74% (n=20), 60% (n=6) and 69% (n=11) remained in the same OCT grade over time in the TLOF, MLOF and TM groups, respectively.

Table 1 summarises the main quantitative retinal imaging findings of each group. ONL thickness was quantified in 28 patients (mean age=20.7), of whom 18 had follow-up data. The mean ONL thickness (µm) at baseline in the TLOF, MLOF and TM groups was 37.07 (range=17–70; SD±15.20), 40.67 (20-59; ±12.53) and 40.38 (14-68; ±18.67), respectively, which was not significantly different (one-way ANOVA; F ratio=0.16; p=0.85). Where further follow-up data were available (mean follow-up time=5.96 years), the mean annual rate of ONL thickness change (µm/year) was −1.29 (range=−2.5 to −0.5; SD±0.9), –1.0 (−2.5 to 0; ±1.08) and −1.29 (−3.0 to 0; ±1.35) for patients in the TLOF (n=7), MLOF (n=4) and TM (n=7) groups, respectively, which was also not significantly different (one-way ANOVA; F ratio=0.098; p=0.91). Subjects in either the TM or MLOF groups with at least one conserved variant (n=6; mean age=23 years; range=4–50; SD±18.74), had a mean baseline ONL thickness of 37.67 µm (range=14–41; SD±13.97) and an annual rate of change of −1.33 µm/year (range=−3.0 to 0; SD±1.33).

EZW loss was quantifiable in 18 patients at baseline (mean age=24.8 years), of whom 14 had follow-up data. The mean EZ loss was 2051 µm (range=538–4318; SD±1318) for patients in the TLOF group (n=13), and 1314 µm (437–2359; ±965) for patients in MLOF (n=4), which was not significantly different (unpaired t-test; p=0.32). Only one patient in the TM group had measurable EZW loss at baseline (OCT grade 3; EZ loss=1181 µm). The mean annual rate of EZ loss (µm/year) was 197.6 (range=10.5–810.5; SD±259) in the TLOF group (n=9), and 58.88 (35.5–90; ±22.8) in the MLOF group (n=4), which was not significantly different (unpaired t-test; p=0.14). The one patient who had measurable EZ loss at baseline in the TM group, had an annual rate of change of 29 µm. Although there is no statistical significance, this may be due to the small sample size. It does reveal a clear trend towards a more severely progressive impairment in retinal architecture in the TLOF group as compared with the other two groups.

### Fundus autofluorescence

**Figure 3:**
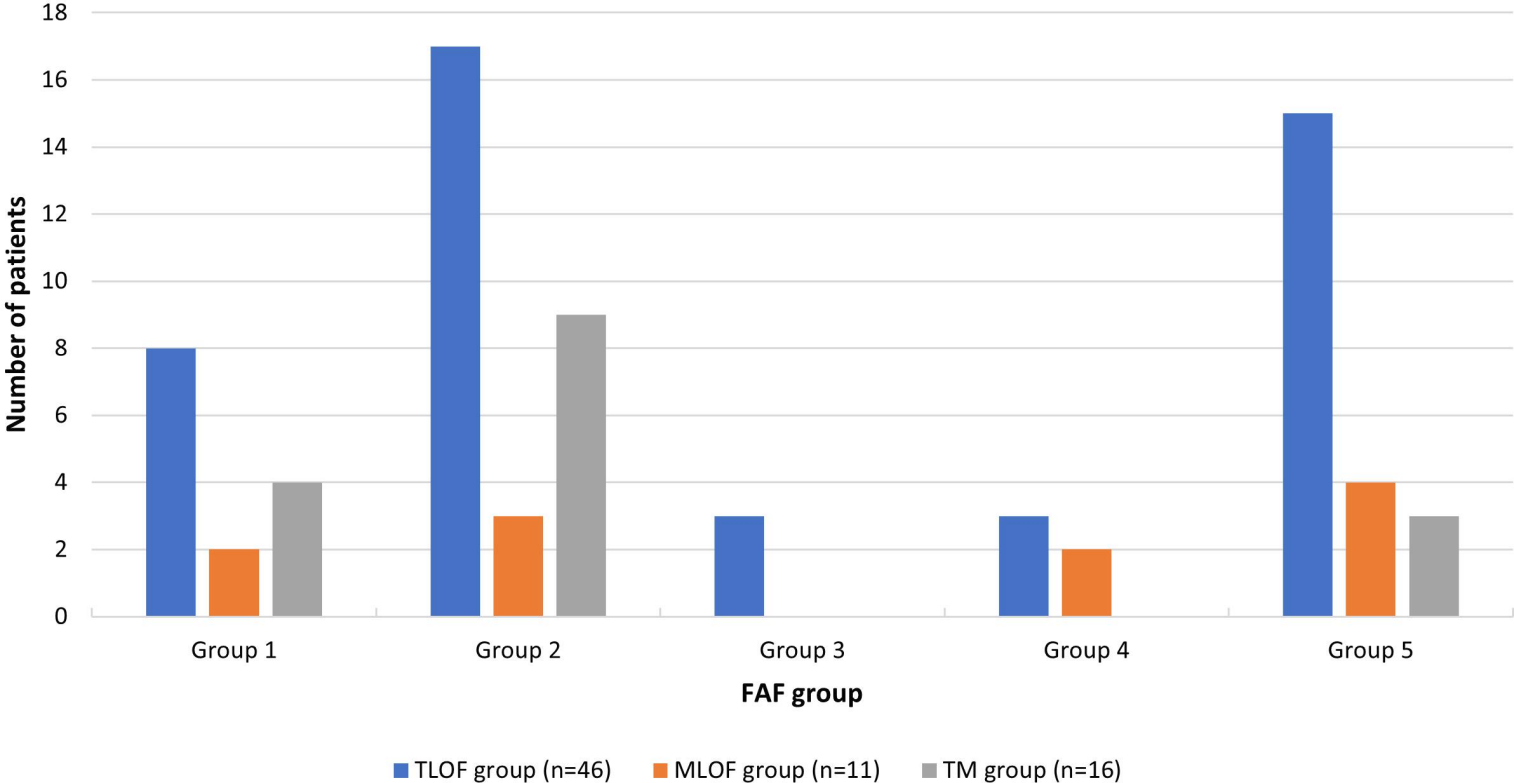
Number of patients in each FAF group at baseline in the TLOF, MLOF and TM groups. Number of patients in each FAF group, with the percentage related to each variant group in total represented on top of each bar. Although the sample size is unequal, it reveals that the proportion of individuals in higher FAF groups was lower when patients had TM. This is in keeping with a more preserved EZ width and better BCVA found in individuals in the TM group, that is, a milder phenotype. BCVA, best-corrected visual acuity; EZ, ellipsoid zone; FAF, fundus autofluorescence; MLOF, missense and one loss-of-function variant; TLOF, two loss-of-function.

Seventy-three patients had baseline FAF (figure 3). In the TLOF group (n=46), 17.4% (n=8) of patients were in FAF group 1, while 39.1% (n=18) were classified as either group 4 or 5, whereas in MLOF (n=11), 18.2% (n=2) were classified in group 1 and 54.5% (n=6) in either group 4 or 5. In patients in the TM group (n=16), 25% (n=4) and 18.8% (n=3) were in FAF group 1, and either 4 or 5, respectively. Forty-one patients had subsequent imaging, with a mean follow-up of 3.65 years (range=0.58–13.75), revealing a change in FAF group in 14.3% (n=3) and 9% (n=1) in TLOF (n=21) and TM (n=11), respectively. No patients in the MLOF (n=8) group had a change in FAF grading. As mentioned in our second report,^3^ only seven patients had quantifiable DAF and/or a ring of increased signal, with a mean age of 39.3 years (range=19.3–59.8), six of whom were in the TLOF group. Overall, this suggests that proportionally, the TM group had more patients with greater structural integrity, which is in keeping with the EZ preservation on OCT and better preserved BCVA in individuals with TM variants. The small number of patients and variability of phenotypes precluded further statistical analysis.

### Electrophysiology

All patients who underwent electrophysiological testing had undetectable PERGs in keeping with severe macular dysfunction, and showed the pathognomonic full-field ERG features of *KCNV2*-retinopathy, described in detail in our first report.^2^

There was a significant negative correlation between DA10 ERG a-waves and age (p=0.01) for the group with TLOF genotypes despite low sample size (n=19), with a mean rate of decline comparable to that seen in the control group. Other ERG components including the DA 0.01 ERG, DA 10 ERG b-wave and LA 30 Hz ERG (amplitudes and peak times) showed no significant correlation with age. There were few subjects in the TM (n=7) and MLOF (n=4) groups, but comparison of ERG component amplitudes and peak times revealed no gross age-associated differences compared with the TLOF cases.

**Figure 4:**
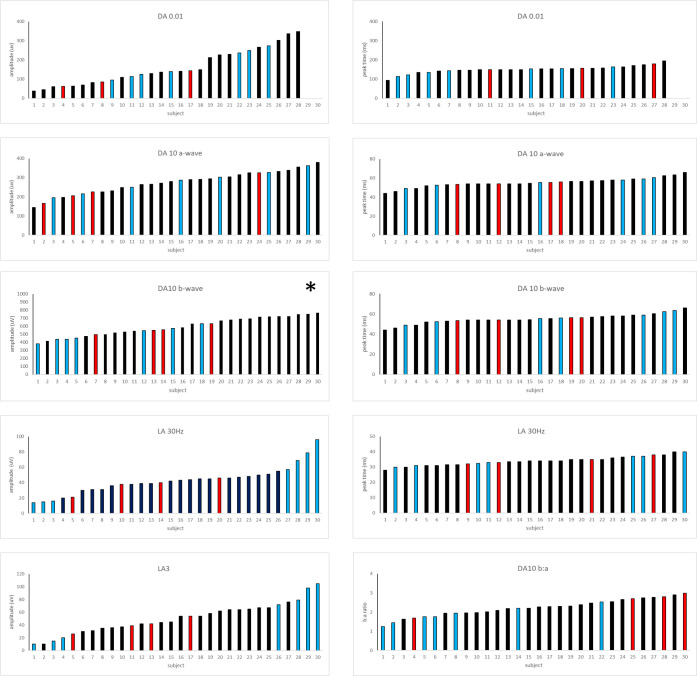
Summary of ERG component peak times and amplitudes. Component peak times and amplitudes for the ISCEV-standard ERGs including dark-adapted (DA) responses to flash strengths of 0.01 cd·s/m^2^ and 10 cd·s/m^2^ (DA0.01 and DA10 ERGs) and light-adapted (LA) responses to a flash strength of 3 cd·s/m^2^ presented at a rate of 2 Hz or 30 Hz (LA3; LA30 Hz). Values are arranged in ascending order for each of the main ERG components and identified according to genotype group; TLOF (black columns; n=19), TM (blue columns; n=7) and MLOF (red columns; n=4). The 11 largest DA10 b-waves (*) were for those in TLOF group and the 4 of 5 lowest in TM group. Patient numbers are not subject-specific and differ between graphs. The graphs highlight the overlap in ERG values associated with each of the three genotype groups. ERG, electroretinography; ISCEV, International Society for Clinical Electrophysiology of Vision; MLOF, missense and one loss-of-function variant; TLOF, two loss-of-function.

There was variation in the DA 10 ERG b-wave amplitudes in the TLOF group, but a trend towards higher values within the reference range. The eleven largest DA10 ERG b-waves (mean 717 uV; n=11) were associated with TLOF genotypes, and 4 of the 5 smallest were associated with TM variants (mean 427 uV; n=4), although intermediate amplitudes were seen for all three groups, highlighting marked overlap. The mean DA 10 ERG b-wave amplitude for all patients within the TLOF, MLOF and TM groups were 636 uV (range 417–768 uV), 557 uV (range 496–632 uV) and 494 uV (range 380–631 uV), respectively. The other main ISCEV standard components showed wide variability within each group, with no obvious trend towards higher or lower values (figure 4).

## Discussion

By using cross-sectional and longitudinal data from the cohorts described in the first two reports of the *KCNV2* retinopathy study group, we have explored possible genotype–phenotype correlations.

The most common type of variant combination in our cohort was TLOF variants—present in approximately 60% (n=55) of the 92 patients included in this report. Our analysis suggests a milder phenotype for patients with TM variants, including better BCVA and greater EZ preservation. The majority of the patients had a disease onset in infancy and at similar ages; even though patients with TLOF variants had the earliest mean age of onset at 3.1 years, this did not reach statistical significance compared with the other groups (p=0.19). Patients with TM variants had an overall statistically significant better BCVA (p=0.03 and 0.035 in right and left eyes, respectively), despite presenting at a similar age. In addition, a smaller proportion of these patients had associated symptoms, such as nyctalopia and photophobia.

Retinal imaging was also in keeping with the above—qualitatively, patients in the TM and MLOF groups had a better baseline OCT and FAF, even when presenting at a similar age, compared with patients in the TLOF group. Quantitatively, the mean baseline ONL thickness was lower in patients with TLOF variants, but the difference was not statistically significant (p=0.85), although interpretation was limited due to the unequal sample size in each group. Likewise, EZW loss was higher in the TLOF than in the MLOF group—of the 18 patients with quantifiable EZW loss at baseline, 13 (72%) had TLOF variants, as opposed to only 1 patient with TM variants. Similarly, the rate of EZW loss (µm/year) was higher in patients with TLOF variants, implying a greater rate of progressive structural damage. This suggests a milder disease course in patients with at least one missense alteration, although the annual rate of ONL thinning appears to be similar between all groups (p=0.91). Interestingly, at baseline, approximately 25% of patients with TM variants had essentially normal retinal autofluorescence, and apart from one subject in the TM group, there were no longitudinal FAF changes in either TM or MLOF. Moreover, most individuals (6/7) with quantifiable FAF parameters, either DAF or ring of increased signal, were in the TLOF group. Of note, patients harbouring variants in conserved protein domains—the vast majority being the common c.1381G>A (p.Gly461Arg) variant, which is localised in the ultra-conserved potassium selective motif (Gly-Tyr-Gly) in the P loop^23 24^—did not have worse BCVA or retinal imaging parameters. These comparisons between conversed domains and other sites may improve our understanding of *KCNV2*-associated retinopathy. However, the small number of patients in either the TM or MLOF group that do not have the common variant precludes additional in-depth interpretation.

Variant severity classifications are not straightforward, and we have not performed protein functional studies to directly ascertain function. Missense alleles have been identified to be markedly deleterious in other genes, such as *ABCA4*, while other deleterious variants such as nonsense variants may not result in a complete lack of function^25^—this may also be the case for certain variants in *KCNV2*. To the best of our knowledge, no hypomorphic allele has been reported to date.

In keeping with the literature, patients showed pathognomonic electrophysiological features of *KCNV2* retinopathy^2 26^ with PERG evidence of severe macular involvement, irrespective of fundus appearance and consistent with previous studies.^2 16 27^ No patient showed a ‘supernormal’ strong flash (DA10) ERG b-wave compared with the upper limit of controls, although the distribution of amplitudes was skewed towards the upper end of the reference range. There was considerable overlap in all of the main ISCEV-standard ERG component amplitudes and peak times in each of the three genotype groups, and although the largest DA 10 ERG b-waves were associated with TLOF, and the smallest with TM variants, assessment of a greater number of patients will be required to fully establish the potential influence of specific *KCNV2* genotypes on quantitative ERG measures of phenotype.

This report represents the largest series to investigate genotype–phenotype correlations in patients with *KCNV2*-retinopathy. However, it has several inherent limitations, mostly due to the multicentre and retrospective nature of the study. There were no standardised protocols for retinal imaging and BCVA. Additionally, statistical analysis was mainly descriptive, since (i) there was wide variability in baseline age and (ii) the sample size was likely not large enough to detect small but potentially significant differences and allow for generalisation of these findings. Long-term, prospective natural history studies with more extensive protocols—including retinal sensitivity measurements—can further characterise the disease and investigate genotype–phenotype correlations.

In summary, in our cohort, patients with missense alterations had a better BCVA and better preservation of retinal architecture, compared with patients harbouring LOF variants. This is of importance for patient prognostication and counselling, as well as patient stratification for future clinical trials.

## Data Availability

Data sharing not applicable as no datasets generated and/or analysed for this study.
